# Synthesis and Insecticidal Activities of New Ester-Derivatives of Celangulin-V

**DOI:** 10.3390/ijms12129596

**Published:** 2011-12-20

**Authors:** Jiwen Zhang, Zhan Hu, Shenkun Li, Wenjun Wu

**Affiliations:** Institute of Pesticide Science, Northwest A&F University, Yangling, Shaanxi 712100, China; E-Mails: nwzjw@yahoo.com.cn (J.Z.); huzhan123cn@yahoo.cn (Z.H.); saintkun001@yeah.net (S.L.)

**Keywords:** *Celastraceae*, dihydroagarofuran, biorational pesticides

## Abstract

In order to develop new biorational pesticides, ten new 6-substituted ester derivatives of Celangulin-V were designed and synthesized. The structures of the new derivatives were confirmed by IR, ^1^H-NMR, ^13^C-NMR and ESI-MS spectral analysis. Insecticidal activities of these compounds were tested against the third-instar larvae of *Mythimna separata*. Two derivatives (**1.1, 1.2**) showed higher insecticidal activities than Celangulin-V, with mortality of 75.0% and 83.3%, respectively. While four compounds (**1.3, 1.4, 1.7, 1.8**) denoted lower insecticidal activities, the others (**1.5, 1.6, 1.9, 1.10**) revealed no activities at a concentration of 10 mg.mL^−1^. The results suggest that C-6 substitutions of Celangulin-V are very important in determining the insecticidal activities of its ester-derivatives. That the acetyl (**1.1**) and propionyl (**1.2**) derivatives possessed much higher insecticidal activities than Celangulin-V itself supported the view that Celangulin-V has the potential to be a lead structure of semi-synthetic green insecticides.

## 1. Introduction

**Figure 1:**
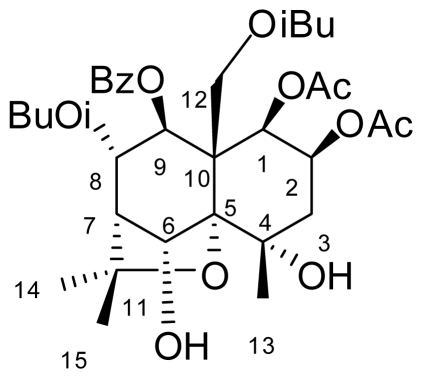
Structure of Celangulin-V.

Plants have been a major source of chemical structure models for insecticides. Several important classes of synthetic insecticides have ancestors in nature. As a result of our program of screening insecticidal activity constituted from plants of China, some sesquiterpene polyol esters with insecticidal activity were isolated [1–4]. Celangulin-V (Figure 1), a β-dihydroagrofuran sesquiterpene polyol ester, from the root bark of Chinese bittersweet, *Celastrus angulatus* Max [5,6], showed prominent stomach toxic effects against *Mythimna separate* acting on mid-gut tissue with a special mechanism of toxicology and suggested it to be a new lead compound for a botanical insecticide [7].

With the objective to search for more potent compounds than Celangulin-V and to clarify the potential structural factors needed for the biological activity of Celangulin-V analogues, ten new C-6 substituted Celangulin-V ester analogues were designed and synthesized. The biological activities of these compounds were tested against the third-instar larvae of *Mythimna separata*.

Like its C-6 ether analogues [8], the insecticidal activities of Celangulin-V C-6 esters varied with the different C-6 substitution. Concurrently, the symptoms of the tested larvae of *Mythimna separata*. were characterized in the same way as in our previous reports[3,4,6–8]. In this paper, the synthesis, characterization and insecticidal activity of new C-6 substituted Celangulin-V esters is described.

## 2. Results and Discussion

### 2.1. Synthesis of Celangulin-V Derivatives

**Scheme 1:**
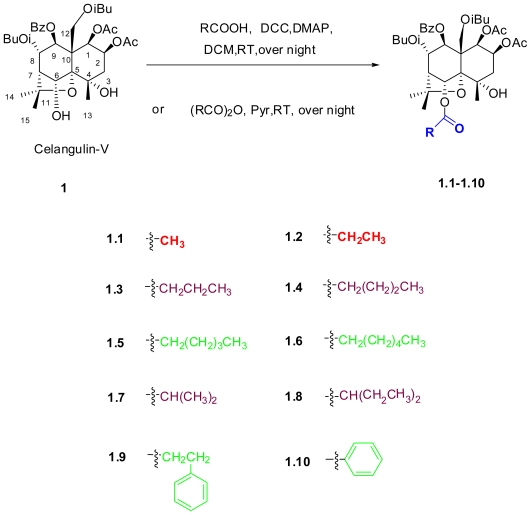
Synthetic route of target compounds. *N*,*N*-dicyclohexylcarbodiimide (DCC),4-dimethylaminopyridine (DMAP), dichloromethane DCM and pyridine (pyr).

As shown in Scheme 1, ten new 6-acyloxy derivatives (**1.1–1.10**) of Celangulin-V were successfully prepared. Compound **1** reacted with anhydrides in dried pyridine at room temperature to give Compounds **1.1, 1.2, 1.3** and **1.7**. Compounds **1.4, 1.5, 1.6, 1.8, 1.9** and **1.10** were obtained from ordinary esterifications of **1** with carboxylic acids by using N,N-dicyclohexylcarbodiimide (DCC) as a condensation agent with a catalytic amount of 4-dimethylaminopyridine (DMAP) at room temperature. The structures of all the target compounds were well characterized by ^1^H-NMR, ^13^C-NMR, ESI-MS, IR, and MP (see Tables 1–3).

### 2.2. Insecticidal Activities

From the insecticidal activities (according to the Table 4), screening, compounds **1.1** and **1.2** exhibited higher activity against 3^th^ instar larvae of *Mythimna separate* than Celangulin-V, compounds(1.3, 1.4, 1.7 and 1.8) exhibited less potency than Celangulin-V, and compounds 1.5, 1.6, 1.9 and 1.10 even no activity at the tested concentration.

## 3. Experimental

### 3.1. General Experimental Procedures

Melting points were measured on an electrothermal digital apparatus made in Beijing and were uncorrected. The ^1^H-NMR (500 MHz), and ^13^C-NMR (125 MHz) were obtained on a Bruker AM-500 FT-NMR spectrometer with CDCl_3_ as the solvent and TMS as the internal standard. MS were recorded under ESI conditions using a Thermo LCQ Fleet instrument. Infrared spectra were measured on a Nicolet FT-IR-20SX instrument using a potassium bromide (KBr) disk, scanning from 625 to 4000 cm^−1^. Optical rotation was measured by Rudolph Autopol II. Analytical thin layer chromatography (TLC) was carried out on precoated plates (silica gel), and spots were visualized with H_2_SO_4_/EtOH. Yields were not optimized. Solvents were dried by standard methods. The title compounds were synthesized under a nitrogen atmosphere.

### 3.2. Synthesis of the Derivatives of Celangulin-V

#### 1β,2β-diacetyloxy-8α,12-diisobutanoyloxy-9β-benzoyloxy-6α-acetyloxy-4α-hydroxy-β-dihydroagarofuran **(1.1):**

A mixture of celangulin-V (**1**) (0.100 g, 0.15 mmol) and acetic anhydride (1 mL) in dry Pyridine (Pyr, 20 mL) was stirred over night at room temperature (RT). When the reaction was complete (checked by TLC), 1mL methanol was added to quench the reaction, then 50mL water was added to the mixture which was then extracted with ethyl acetate (50 mL × 3). The ethyl acetate layers were combined and washed with 30mL water and 5mL saturated sodium chloride, dried over anhydrous sodium sulfate and separated by column chromatography(silica gel, 200~300 mesh) with a gradient of petroleum ether (60–90 °C) and ethyl acetate as eluent to yield compound **1.1** as white powder.ESI-MS: Found 727 [M + Na]^+^. Its IR spectrum revealed characteristic ester absorptions at 1722 cm^−1^, and a free hydroxyl absorption at 3552 cm^−1^. (6-)OCOCH_3_ was certified by ^1^H-NMR δ: 2.13 (s, 3H) and ^13^C-NMR δ: 169.58 (CO), 21.42 (CH_3_), other data see Table 2 and Table 3.

#### 1β,2β-diacetyloxy-8α,12-diisobutanoyloxy-9β-benzoyloxy-6α-propyloxy-4α-hydroxy-β-dihydroagarofuran **(1.2):**

This compound was obtained as a white powder following a similar procedure to the synthesis of compound 1.1. ESI-MS: Found 741 [M + Na]^+^. Its IR spectrum revealed characteristic ester absorptions at 1726 cm^−1^, and a free hydroxyl absorption at 3548 cm^−1^. (6-)OCOCH_2_CH_3_ was certificated by ^1^H-NMR δ: 1.19 (3H, t, 7.5), 2.42 (2H, q, 7.5) and ^13^C-NMR δ: 172.84 (CO), 28.21 CH_2_, 8.95 CH_3_, other data see Table 2 and Table 3.

#### 1β,2β-diacetyloxy-8α,12-diisobutanoyloxy-9β-benzoyloxy-6α-butanoyloxy-4α-hydroxy-β-dihydroagarofuran **(1.3):**

This compound was obtained as a white powder following a similar procedure to the synthesis of the compound 1.1. ESI-MS: Found 755 [M + Na]^+^. Its IR spectrum revealed characteristic ester absorptions at 1721 cm^−1^, and a free hydroxyl absorption at 3546 cm^−1^. (6-)OCOCH_2_CH_2_CH_3_ was certificated by ^1^H-NMR δ: 2.35 (m, 2H), 1.70 (m, 2H), 0.96 (3H, t, 7.0) and ^13^C-NMR δ: 172.07 (CO), 18.16 CH_2_, 36.75 CH_2_, 13.73 CH_3_, other data see Table 2 and Table 3.

#### 1β,2β-diacetyloxy-6α,8α,12-triisobutanoyloxy-9β-benzoyloxy-4α-hydroxy-β-dihydro-agarofuran **(1.7):**

This compound was obtained as a white powder following a similar procedure to the synthesis of the compound 1.1. ESI-MS: Found 755 [M + Na]^+^. Its IR spectrum revealed characteristic ester absorptions at 1724 cm^−1^, and a free hydroxyl absorption at 3542 cm^−1^. (6-)OCOCH(CH_3_)_2_ was certificated by ^1^H-NMR δ: 1.21 (d, 3H, 7.0), 1.22 (d, 3H, 7.0), 2.59 (m, 1H) and ^13^C-NMR δ: 177.00 (CO), 34.42 CH, 18.99 CH_3_, 19.14 CH_3_, other data see Table 2 and Table 3.

#### 1β,2β-diacetyloxy-8α,12-diisobutanoyloxy-9β-benzoyloxy-6α-pentanoyloxy-4α-hydroxy-β-dihydroagarofuran **(1.4):**

A mixture of celangulin-V (**1**) (0.100 g, 0.15 mmol), pentanoic acid (1 mL), N,N-dicyclohexylcarbodiimide (DCC, 0.062 g, 0.30 mmol) and dimethylaminopyridine (DMAP, 0.005 g, 0.04 mmol) in dry dichloromethane (DCM 20 mL) was stirred over night at room temperature. When the reaction was complete (checked by TLC), 1 mL methanol was added to quench the reaction. Then 30mL water was added to the mixture and the resulting suspension was filtered then extracted with dichloromethane (30 mL × 3). The dichloromethane layers were combined and washed with 30mL water and 5mL saturated sodium chloride, dried over anhydrous sodium sulfate and separated by column chromatography(silica gel, 200~300 mesh) with a gradient of petroleum ether (60–90 °C) and ethyl acetate as eluent to yield compound **1.4** as a white powder. ESI-MS: Found 769 [M + Na]^+^. Its IR spectrum revealed characteristic ester absorptions at 1718 cm^−1^, and a free hydroxyl absorption at 3547 cm^−1^. (6-)OCOCH_2_CH_2_CH_2_CH_3_ was certificated by ^1^H-NMR δ: 0.92 (m, 3H), 1.33 (m, 2H), 2.38 (m, 2H), 1.65 (m, 2H) and ^13^C-NMR δ: 172.24 (CO), 13.66 CH_3_, 22.27 CH_2_, 26.68 CH_2_, 34.58 CH_2_, other data see Table 2 and Table 3.

#### 1β,2β-diacetyloxy-8α,12-diisobutanoyloxy-9β-benzoyloxy-6α-hexanoyloxy-4α-hydroxy-β-dihydroagarofuran **(1.5):**

This compound was obtained as a white powder following a similar procedure to the synthesis of the compound 1.4. ESI-MS: Found 783 [M + Na]^+^. Its IR spectrum revealed characteristic ester absorptions at 1718 cm^−1^, and a free hydroxyl absorption at 3543 cm^−1^. (6-)OCOCH_2_CH_2_CH_2_CH_2_CH_3_ was certificated by ^1^H-NMR δ: 0.88 (t, 3H, 7.0), 1.31 (m, 4H), 2.38 (m, 2H), 1.68 (m, 2H) and ^13^C-NMR δ: 172.07 (CO), 22.24 CH_2_, 24.31 CH_2_, 31.26 CH_2_, 34.81 CH_2_, 13.87 CH_3_, other data see Table 2 and Table 3.

#### 1β,2β-diacetyloxy-8α,12-diisobutanoyloxy-9β-benzoyloxy-6α-heptanoyloxy-4α-hydroxy-β-dihydroagarofuran **(1.6):**

This compound was obtained as a colorless oil following a similar procedure to the synthesis of the compound 1.4.ESI-MS: Found 797 [M + Na]^+^. Its IR spectrum revealed characteristic ester absorptions at 1716 cm^−1^, and a free hydroxyl absorption at 3547 cm^−1^. (6-)OCOCH_2_CH_2_CH_2_CH_2_CH_2_CH_3_ was certificated by ^1^H-NMR δ: 0.88 (t, 3H, 7.0), 1.29 (m, 6H), 2.36 (m, 2H), 1.66 (m, 2H) and ^13^C-NMR δ: 172.26 (CO), 22.47 CH_2_, 24.61 CH_2_, 28.82 CH_2_, 31.37 CH_2_, 34.86 CH_2_, 14.01 CH_3_, other data see Table 2 and Table 3.

#### 1β,2β-diacetyloxy-8α,12-diisobutanoyloxy-9β-benzoyloxy-6α-(2-ethylbutanoyloxy)-4α-hydroxy-β-dihydro-agarofuran **(1.8):**

This compound was obtained as a white powder following a similar procedure to the synthesis of the compound 1.4. ESI-MS: Found 783 [M + Na]^+^. Its IR spectrum revealed characteristic ester absorptions at 1720 cm^−1^, and a free hydroxyl absorption at 3544 cm^−1^. (6-)OCOCH(CH_2_CH_3_)_2_ was certificated by ^1^H-NMR δ: 2.28 (1H, m), 1.70 (4H, m), 0.91 (6H, m) and ^13^C-NMR δ: 174.66 (CO), 49.29 CH, 24.58 CH_2_, 24.25 CH_2_, 12.02 CH_3_, 11.88 CH_3_, other data see Table 2 and Table 3.

#### 1β,2β-diacetyloxy-8α,12-diisobutanoyloxy-9β-benzoyloxy-6α-(3-phenylpropanoyloxy)-4α-hydroxy- β-dihydro-agarofuran **(1.9):**

This compound was obtained as a white powder following a similar procedure to the synthesis of the compound 1.4. ESI-MS: Found 817 [M + Na]^+^. Its IR spectrum revealed characteristic ester absorptions at 1716 cm^−1^, and a free hydroxyl absorption at 3547 cm^−1^. (6-)OCOCH_2_CH_2_C_6_H_5_ was certificated by ^1^H-NMR δ: 2.72 (m, 2H), 3.00 (m, 2H), 7.29 (m, 2H), 7.20 (m, 3H) and ^13^C-NMR δ: 171.45 (CO), 36.36 CH_2_, 30.68 CH_3_, 140.05 C, 126.41 CH, 128.57 CH × 2, 128.31 CH × 2, other data see Table 2 and Table 3.

#### 1β,2β-diacetyloxy-8α,12-diisobutanoyloxy-6α,9β-dibenzoyloxy-4α-hydroxy-β-dihydroagarofuran **(1.10):**

This compound was obtained as a white powder following a similar procedure to the synthesis of the compound 1.4. ESI-MS: Found 789 [M + Na]^+^. Its IR spectrum revealed characteristic ester absorptions at 1722 cm^−1^, and a free hydroxyl absorption at 3543 cm^−1^. (6-)OCOC_6_H_5_ was certificated by ^1^H-NMR δ: 8.19 (2H, m), 7.47 (2H, m), 7.58 (1H, m) and ^13^C-NMR δ: 165.48 (CO), 128.67 (2 × CH), 133.46 CH, 130.18 (2 × CH), 129.64 C, other data see Table 2 and Table 3.

### 3.3. Bioassay for Insecticidal Activities

Wheat leaf discs of known area (0.5 cm × 0.5 cm) were treated with 1μL from 10 mg test samples dissolved in 1mL acetone (acetone and celangulin V were used as negative and positive controls). The 3^rd^ instar larvae of *Mythimna separate* were fed with the discs over 2 h (repeated 3 times for each sample). After 2 h, the numbers of knocked down larvae (symptoms: the larvae were narcotized, the bodies were very soft and immobilized, and response disappeared completely) were recorded. [3]. The insecticidal activity result can be found in Table 4.

## 4. Conclusions

In conclusion, ten new 6-acyloxy derivatives of Celangulin-V (**1.1–1.10**) have been synthesized and evaluated for their insecticidal activities against the 3^rd^ instar larvae of *Mythimna separate* in *vivo* at a concentration of 10 mg.mL^−1^. The bioassay of these analogues showed that some synthesized ester-derivatives of Celangulin-V showed the same stomach toxic effects against *Mythimna separate* as Celangulin-V and that the poisoned insects had the same symptoms as from its ether-derivatives. This implies the insecticidal ester-derivatives may act on the same target protein as Celangulin-V. The substitutions of C-6 have a notable influence on the insecticidal activity. Among the Celangulin-V ester-derivatives, two and three carbon substitutions may be more favorable for promoting the insecticidal activity as compared with much larger substitution groups at the C-6 position. These results have encouraged us to start further investigations into novel Celangulin-V derivatives as insecticidal agents and this will be reported in due course.

## Figures and Tables

**Table 1:** Experimental Data of Celangulin-V and its derivatives.

Compounds	Molecular Formula	M.P °C	ESI-MS (M + Na)	Optical Rotation [α]°, (MeOH, c = mg.100 mL^−1^)
**Celangulin-V**	C_34_H_46_O_13_	198–200	685	−12.2, (10.8)
**1.1**	C_36_H_48_O_14_	214–216	727	−10.6, (16.0)
**1.2**	C_37_H_50_O_14_	94–96	741	−10.1, (15.0)
**1.3**	C_38_H_52_O_14_	114–116	755	−10.3, (18.5)
**1.4**	C_39_H_54_O_14_	102–104	769	−33.0, (18.0)
**1.5**	C_40_H_56_O_14_	64–66	783	−15.2, (15.0)
**1.6**	C_41_H_58_O_14_	colorless oils	797	−26.4, (19.0)
**1.7**	C_38_H_52_O_14_	98–100	755	−39.6, (17.0)
**1.8**	C_40_H_56_O_14_	112–114	783	−95.8, (11.5)
**1.9**	C_43_H_54_O_14_	124–126	817	−29.7, (12.5)
**1.10**	C_41_H_06_O_14_	96–98	789	−51.7, (13.5)

**Table 2:** ^13^C-NMR Spectral Data [125 MHz, δ (ppm)] for compounds **1 and 1.1–10** in CDCl_3_.

No.	1	1.1	1.2	1.3	1.4	1.5	1.6	1.7	1.8	1.9	1.10
1	75.17	75.20	75.22	75.22	75.23	75.22	75.23	75.23	75.25	75.19	75.20
2	67.44	67.69	67.71	67.70	67.72	67.71	67.72	67.71	67.72	67.68	67.74
3	41.25	42.01	41.98	41.94	41.94	41.93	41.94	41.99	41.79	41.99	42.51
4	72.23	69.61	69.64	69.63	69.63	69.63	69.65	69.64	69.64	69.59	69.81
5	91.56	92.09	92.11	92.08	92.08	92.08	92.08	92.17	92.04	92.08	91.94
6	77.04	75.60	75.58	75.59	75.60	75.60	75.60	75.61	75.69	75.63	76.43
7	53.69	52.31	52.36	52.40	52.39	52.39	52.39	52.43	52.62	52.31	52.31
8	73.86	75.19	73.57	73.54	73.55	73.54	73.55	73.60	73.48	73.51	73.39
9	75.45	75.54	75.49	75.46	75.49	75.49	75.50	75.42	75.69	75.53	75.70
10	50.71	51.46	51.47	51.47	51.48	51.47	51.48	51.49	51.54	51.45	51.57
11	84.61	84.34	84.31	84.29	84.29	84.28	84.29	84.29	84.21	84.27	84.44
12	61.82	61.79	61.80	61.79	61.80	61.80	61.81	61.79	61.79	61.79	61.87
13	24.32	24.85	24.93	24.93	24.96	24.97	24.98	24.92	25.13	24.76	24.57
14	26.45	25.89	25.90	25.89	25.90	25.90	25.91	25.89	25.87	25.86	25.89
15	30.15	29.72	29.72	29.73	29.74	29.74	29.75	29.75	29.83	29.66	29.81

**Table 3:** ^1^H-NMR Spectral Data [500 MHz, δ_H_ (*J* in Hz)] of compounds **1 and 1.1–10** in CDCl_3_.

No.	1	1.1	1.2	1.3	1.4	1.5	1.6	1.7	1.8	1.9	1.10
1	5.49 (d,1H, 3.5)	5.48 (d,1H, 3.5)	5.48 (d,1H, 3.5)	5.48 (d,1H, 3.5)	5.48 (d,1H, 3.5)	5.48 (d,1H, 3.5)	5.48 (d,1H, 3.5)	5.48 (d,1H, 3.5)	5.49 (d,1H, 3.5)	5.46 (d,1H, 3.5)	5.52 (d,1H, 3.5)
2	5.39 (m,1H)	5.36 (m,1H)	5.36 (m,1H)	5.36 (m,1H)	5.36 (m,1H)	5.36 (m,1H)	5.36 (m,1H)	5.36 (m,1H)	5.37 (m,1H)	5.35 (m,1H)	5.39 (m,1H)
3	2.09 (m,1H), 2.03 (m,1H)	2.11 (m,1H), 1.97 (m,1H)	2.11 (m,1H), 1.96 (m,1H)	2.12 (m,1H), 2.07 (m,1H)	2.14 (m,1H), 2.09 (m,1H)	2.12 (m,1H), 1.95 (m,1H)	2.12 (m,1H), 1.95 (m,1H)	2.13 (m,1H), 1.95 (m,1H)	2.12 (m,1H), 1.95 (m,1H)	2.11 (m,1H), 1.95 (m,1H)	2.17 (m,1H), 2.02 (m,1H)
6	5.22(s, 1H)	6.48(s, 1H)	6.49(s, 1H)	6.49(s, 1H)	6.48(s, 1H)	6.48(s, 1H)	6.48(s, 1H)	6.49(s, 1H)	6.48(s, 1H)	6.47(s, 1H)	6.61(s, 1H)
7	2.58 (d,1H, 3.5)	2.49 (d,1H, 3.5)	2.48 (d,1H, 3.5)	2.47 (d,1H, 3.5)	2.47 (d,1H, 3.5)	2.47 (d,1H, 3.5)	2.47 (d,1H, 3.5)	2.46 (d,1H, 3.5)	2.43 (d,1H, 3.5)	2.39 (d,1H, 3.5)	2.67 (d,1H, 3.5)
8	5.62 (dd,1H, 9.5,3.5)	5.77 (dd,1H, 9.5,3.5)	5.78 (dd,1H, 9.5,3.5)	5.79 (dd,1H, 9.5,3.5)	5.78 (dd,1H, 9.5,3.5)	5.79 (dd,1H, 9.5,3.5)	5.78 (dd,1H, 9.5,3.5)	5.79 (dd,1H, 9.5,3.5)	5.81 (dd,1H, 9.5,3.5)	5.76 (dd,1H, 9.5,3.5)	5.93 (dd,1H, 9.5,3.5)
9	6.06 (d,1H, 9.5)	6.03 (d,1H, 9.5)	6.04 (d,1H, 9.5)	6.04 (d,1H, 9.5)	6.04 (d,1H, 9.5)	6.03 (d,1H, 9.5)	6.03 (d,1H, 9.5)	6.04 (d,1H, 9.5)	6.03 (d,1H, 9.5)	6.02 (d,1H, 9.5)	6.09 (d,1H, 9.5)
12	4.87, 4.65 (Abq, 2H, 13.5)	4.90, 4.65 (Abq, 2H, 13.5)	4.90, 4.65 (Abq, 2H, 13.5)	4.90, 4.64 (Abq, 2H, 13.5)	4.90,4.64 (Abq,2H, 13.5)	4.90, 4.64 (Abq, 2H, 13.5)	4.890, 4.64 (Abq, 2H, 13.5)	4.89, 4.65 (Abq, 2H, 13.5)	4.91, 4.64 (Abq, 2H, 13.5)	4.88, 4.64 (Abq, 2H, 13.5)	4.93, 4.70 (Abq, 2H, 13.5)
13	1.77(s, 3H)	1.57(s, 3H)	1.57(s, 3H)	1.73(s, 3H)	1.73(s, 3H)	1.74(s, 3H)	1.72(s, 3H)	1.72(s, 3H)	1.72(s, 3H)	1.69(s, 3H)	1.75(s, 3H)
14	1.72(s, 3H)	1.72(s, 3H)	1.72(s, 3H)	1.57(s, 3H)	1.57(s, 3H))	1.56(s, 3H)	1.56(s, 3H)	1.58(s, 3H)	1.58(s, 3H)	1.47(s, 3H)	1.59(s, 3H)
15	1.61(s, 3H)	1.50(s, 3H)	1.49(s, 3H)	1.49(s, 3H)	1.49(s, 3H)	1.49(s, 3H)	1.49(s, 3H)	1.49(s, 3H)	1.51(s, 3H)	1.42(s, 3H)	1.53(s, 3H)

**Table 4:** Insecticidal activities of Celangulin-V derivatives.

Compounds	Mortality(%)
Acetone	0
Celangulin-V	66.7
1.1	**75.0**
1.2	**83.3**
1.3	16.7
1.4	33.3
1.5	0
1.6	0
1.7	8.3
1.8	8.3
1.9	0
1.10	0

Note: The concentration is a 10 mg test sample dissolved in 1 mL acetone.
